# Transcriptomics of chicken cecal tonsils and intestine after infection with low pathogenic avian influenza virus H9N2

**DOI:** 10.1038/s41598-021-99182-3

**Published:** 2021-10-14

**Authors:** Nadiyah Alqazlan, Mehdi Emam, Éva Nagy, Byram Bridle, Mehdi Sargolzaei, Shayan Sharif

**Affiliations:** 1 Department of Pathobiology, Ontario Veterinary College, University of Guelph, Guelph, ON N1G 2W1 Canada; 2 Department of Human Genetics, McGill University, Montreal, QC H3A 0E7 Canada; 3 Select Sires, Inc., Plain City, OH 43064 USA

**Keywords:** Innate immunity, Influenza virus, Chemokines, Cytokines, Infection, RNA sequencing

## Abstract

Influenza viruses cause severe respiratory infections in humans and birds, triggering global health concerns and economic burden. Influenza infection is a dynamic process involving complex biological host responses. The objective of this study was to illustrate global biological processes in ileum and cecal tonsils at early time points after chickens were infected with low pathogenic avian influenza virus (LPAIV) H9N2 through transcriptome analysis. Total RNA isolated from ileum and cecal tonsils of non-infected and infected layers at 12-, 24- and 72-h post-infection (hpi) was used for mRNA sequencing analyses to characterize differentially expressed genes and overrepresented pathways. Statistical analysis highlighted transcriptomic signatures significantly occurring 24 and 72 hpi, but not earlier at 12 hpi. Interferon (IFN)-inducible and IFN-stimulated gene (ISG) expression was increased, followed by continued expression of various heat-shock proteins (HSP), including HSP60, HSP70, HSP90 and HSP110. Some upregulated genes involved in innate antiviral responses included *DDX60*, *MX1*, *RSAD2* and *CMPK2*. The ISG15 antiviral mechanism pathway was highly enriched in ileum and cecal tonsils at 24 hpi. Overall, most affected pathways were related to interferon production and the heat-shock response. Research on these candidate genes and pathways is warranted to decipher underlying mechanisms of immunity against LPAIV in chickens.

## Introduction

Avian influenza viruses (AIVs) pose major public health threats and significant economic losses to the poultry industry^1,2^. AIVs possess an error-prone RNA polymerase making them susceptible to reassortment, which occasionally results in the emergence of zoonotic viruses^3,4^. Low pathogenic (LP) AIVs of subtype H9N2 are widespread pathogens of poultry. They are listed by the World Health Organization as a zoonotic influenza virus and ranked by Centers for Disease Control and Prevention (CDC) as potential pandemic viruses^5,6^. A novel influenza A (H7N9) virus is likely to have acquired six viral segments from circulating avian H9N2 viruses^7^.

The subtype of the virus is a critical factor in understanding the host’s ability to regulate the immune responses against AIV^8–11^. LPAIVs principally replicate in the avian respiratory and gastrointestinal tract (GIT), causing mild signs of illness^12^. Replication of LPAIVs in the GIT disrupts the gut microbiota and dysregulates type I interferons (IFN)^13–15^. The GIT plays a critical role in immune homeostasis, balancing the protection of the host against invading pathogens and interacting with commensal microbiota^16–18^. The well-developed gut-associated lymphoid tissues in the GIT are the first line of defence against invading pathogens and consist of various lymphoid cells found in Peyer’s patches, cecal tonsils and epithelial lining and lamina propria of the GIT^19^. Peyer’s patches are located at the anti-mesenterial side of the chicken jejunum, and one Peyer’s patch is consistently found in ileum at the proximal ileocecal transition^20^. Peyer’s patches epithelium has specialized cells called M cells that overlay follicles of defined T and B cell areas. Cecal tonsils are located at the cecum–rectum junction and have a structure similar to Peyer’s patches^20,21^. Antigen recognition through M cells or by direct antigen sampling by macrophages and dendritic cells induce intestinal immune responses^22,23^.

There has been significant interest in understanding how influenza viruses interact with their host. To this end, several studies have interrogated transcriptomics of host responses to LPAIV and highly pathogenic AIV^24–26^. These studies have revealed a complex network of immune responses initiated by avian influenza viruses, at cellular and tissue levels, in the respiratory tract^24,27–30^, ileum^8,31^, dendritic cells^25^ and chicken embryonic fibroblasts^32^. It has become evident that avian influenza viruses trigger the innate receptors of their host. Pattern-recognition receptors (PRRs) are the host's innate immune sensors, and at least three distinct classes of PRRs recognize influenza virus infection^33,34^. This recognition leads to secretion of cytokines, chemokines and antimicrobial peptides by cells, including dendritic cells and macrophages. Additional immune responses occur at immunological induction sites, including activation of IFN-stimulated genes (ISG)^35,36^. Remarkably, viral mechanisms antagonizing Toll-like receptor-mediated signaling by influenza viruses have not been described^37^. However, the influenza virus non-structural 1 (NS1) protein limits type I IFN responses. Deletions in influenza virus NS1 provoke augmented caspase‐1 activity post-infection of macrophages resulting in enhanced secretion of interleukin (IL)‐1β and IL-18, which indicates an antagonizing function of NS1^37^. Viral NS1 also targets melanoma differentiation-associated protein 5 (MDA5) and directly interferes with the function of different ISGs^37,38^.

The increasing appreciation of the complexity of GIT immune responses has increased research efforts to decipher the mechanisms behind their induction and regulation. However, there is still a paucity of information about underlying mechanisms of chicken immunity against pathogens, including AIV in the GIT. The largest lymphoid aggregates of avian gut-associated lymphoid tissue are cecal tonsils. Along with Peyer's patches in the intestine, cecal tonsils elicit protective immune responses against bacterial and viral pathogens in avian species. Therefore, the present study was designed to examine gene expression in ileum and cecal tonsils, as potential sites for virus replication and induction of immune responses. Our findings could facilitate the development of effective measures to enhance immunity against AIV in poultry.

## Results

### RNA-seq data analysis

The average number of reads that passed the quality control was 23.7 and 25.5 million reads per sample for cecal tonsils and ileum, respectively. The average percentage of reads uniquely mapped to the chicken reference genome was 89.7% and 91.7% of the reads for cecal tonsils and ileum, respectively.

### Global profiles of DE genes in chicken infected with low pathogenic AIV subtype H9N2

#### Transcriptomic changes were detectable in cecal tonsils and peaked at 24 hpi

**Figure 1:**
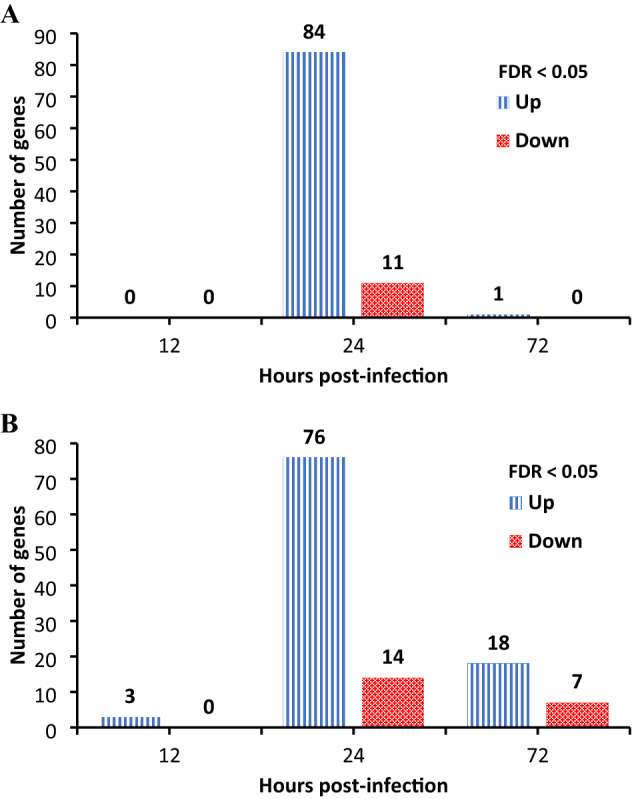
The number of significantly differentially expressed genes in the cecal tonsils and the ileum. Significantly differentially expressed genes in the cecal tonsils (panel A) and the ileum (panel B) following influenza virus infection (significant p-value of False Discovery Rate (FDR) controlled at 5%) over time. Blue bars indicate the number of upregulated genes, and the red bars indicate the number of downregulated genes.

**Figure 2:**
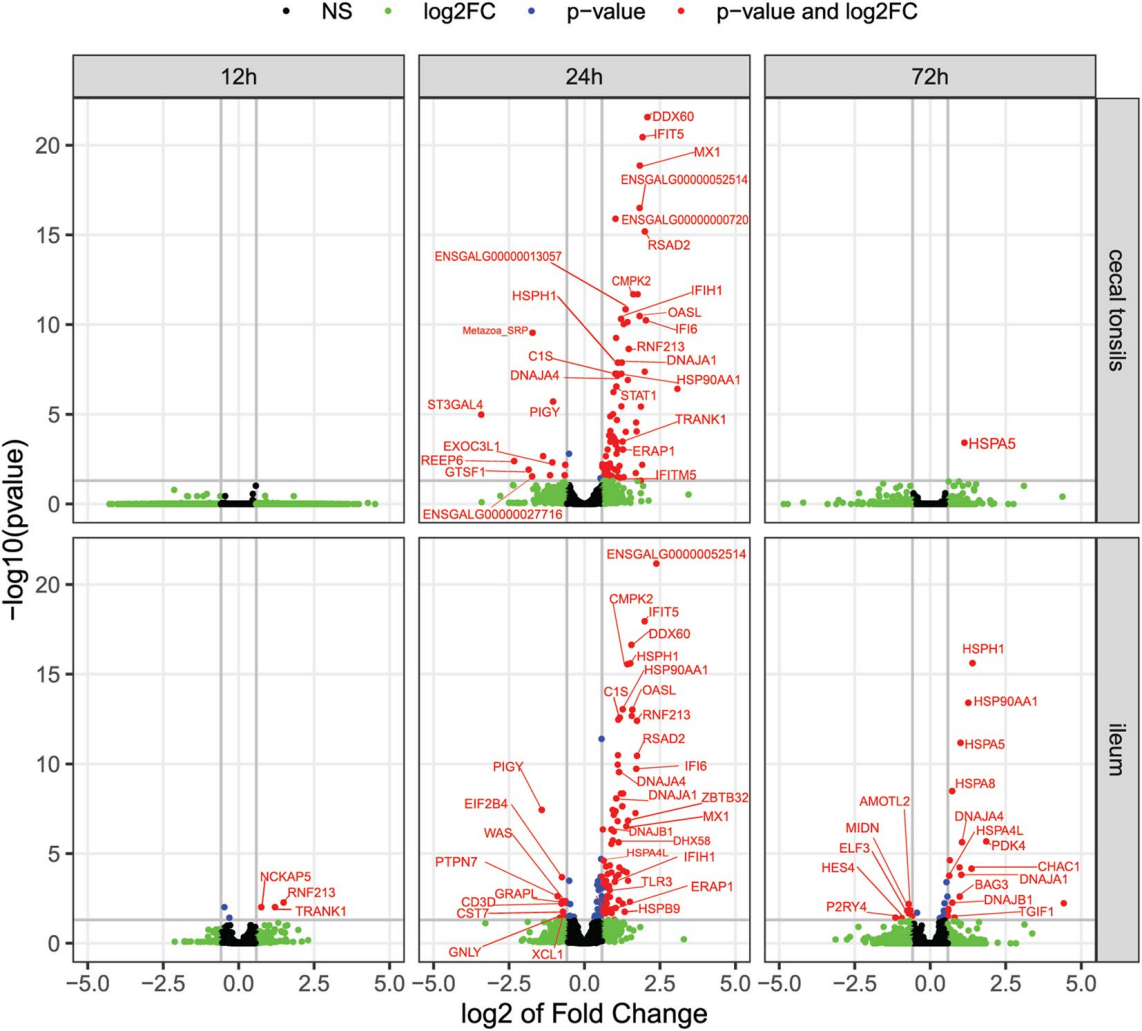
Volcano plots showing changes in gene expression. Volcano plots showing changes in the expression of individual genes over time (12, 24, and 72 h post-infection) in the cecal tonsils and the ileum. The number of genes decreased linearly as the fold change increased. Genes with p-values of False Discovery Rate at < 5% and fold change of > 1.5 or < -− 1.5 are depicted in red.

Comparing the gene expression in cecal tonsil tissues between infected and non-infected groups, the highest number of DE genes was 24 hpi and reduced to near baseline at 72 hpi (Table 1; Fig. 1A). The significant changes were also illustrated for DE genes with volcano plots (Fig. 2).

**Table 1:** Significant gene expression changes following influenza infection.

	Tissue	Differentially expressed genes		
		12 h post-infection	24 h post-infection	72 h post-infection
Number of DE genes FC > 1.5	Cecal tonsils	0	95	1
		0	84↑;11↓	1↑
	Ileum	3	90	25
		3↑	76↑;14↓	18↑;7↓
Highest upregulated gene: Description	Cecal tonsils	–	ENSGALG00000039205 Novel gene T-complex protein 1 subunit gamma-like	ENSGALG00000001000 /HSPA5
	Ileum	ENSGALG00000039269/ *RNF213* ring finger protein 213	ENSGALG00000052514 Novel gene early endosome antigen 1-like	ENSGALG00000049807 Novel gene
Highest downregulated gene: Description	Cecal tonsils	–	ENSGALG00000029747 /*ST3GAL4*	–
	Ileum	–	ENSGALG00000026229/*PIGY* phosphatidylinositol glycan anchor biosynthesis class Y	ENSGALG00000004346/*P2RY4* pyrimidinergic receptor P2Y4

Cecal tonsils, ileum. FC, fold change; ↑, upregulated; ↓, downregulated.

At 12 hpi (Fig. 1A), there were no DE genes between AIV-infected and non-infected chickens’ cecal tonsil tissues (FDR < 5%). However, using FC-based significance (FC < 1.5), we found a downregulated gene (*FGF19*), which is involved in mitogen-activated protein kinase (MAPK)1/MAPK3 signaling and cytokine signaling in immune system pathways.

Changes in DE genes began in cecal tonsils at 24 hpi (Fig. 1A). Ninety-five genes had a significant fold-change in expression (84 upregulated, 11 downregulated) (Table 1). The fold-change of gene expression between infected and non-infected groups ranged from 7.27 to -3.41. The gene with the highest upregulation was a novel gene called T-complex protein 1 subunit gamma-like. This gene is an orthologue of turkey’s *CCT3* (Chaperonin Containing T-complex protein 1 subunit 3) gene and encodes for a chaperonin member containing TCP1 complex. T-complex protein 1 (TCP1) is a chaperonin class of protein involved in intracellular assembly and folding of various proteins. Specifically, this protein’s gamma subunit plays a crucial role in folding and assembling cytoskeleton proteins, exploited by viruses during early viral infection. TCP1 is also a cytosolic HSP60 family protein. Another gene with significant upregulation was the early endosome antigen 1-like, a novel long non-coding RNA (lncRNA). We also found upregulation of HSPs (HSP90AA1, HSPH1, HSPA8) and some genes involved in innate immune and antiviral responses, including IFN-inducible genes and ISGs (*DDX60*, *IFI6*, *RSAD2*, *IFIT5, MX1, OASL*, *CMPK2*, *RNF213*, *ERAP1*, *IFIH1*, *STAT1*, *DHX58*, *IFITM5* (interferon-induced transmembrane) and *C1S* (complement C1s)). The *MX1* gene prevents the import of nuclear material into viral nucleocapsids, which is known to have a disruptive effect on influenza A viruses by disrupting lipid rafts of the virus. *CMPK2* is involved in the differentiation of monocytes and is involved in metabolism. The *ERAP1* gene is involved in class I major histocompatibility complex (MHC)-mediated antigen processing and presentation. The *DNAJA1* gene is an important paralog of *DNAJA4*, and response to heat and protein folding are some of its related annotations (GO:0,009,408, GO:0,006,457). *C1S* was an upregulated gene in the present study and is involved in the innate immune system and regulation of complement cascade pathways. A novel chicken gene characterized as an orthologue to human *USP18*, was upregulated. This novel chicken gene is involved in interferon signaling, antiviral mechanisms by IFN-stimulated genes and ISG15. Another novel chicken gene, described as guanylate binding protein 1, was also upregulated and among its GO term was cellular response to interferon-gamma. Downregulated genes in cecal tonsils at 24 hpi included a novel gene (an orthologue of human *SAA* gene). This gene is expressed in granulocytes and involved in the biological process of acute-phase responses (GO:0,006,953, acute inflammatory response to infection) and the innate immune system pathway (R-GGA-168249).

At 72 hpi, only *HSPA5* had a significant upregulated expression (FDR < 5%) (Fig. 1A; Table 1). The FDR threshold was set to 10% to examine genes under a relaxed level of significance. Five HSPs were significantly different at this level. Additionally, one of these upregulated genes was *CIDEC* (with the greatest FC > 1.5). *CIDEC* (cell death-inducing DFFA (DNA fragmentation factor-alpha)-like effector c) is involved in regulating the apoptotic process. The *DOP1B* gene was among the results for both FC > 1.5 and FDR < 10% and is known to increase during responses to infections.

#### Transcriptomic changes were detected in the ileal tissue at 12 hpi and peak at 24 hpi

Differential gene expression in ileal tissue samples of infected chickens was detected as early as 12 hpi, and further increased at 24 hpi and then decreased 72 hpi (Fig. 1B; Table 1). This changing dynamic and the number of significant changes were somewhat different from the cecal tonsil samples; the number of DE genes at 72 hpi was maintained above ileum baseline. The differences in DE genes and changing dynamics for each time point post-infection were further illustrated in volcano plots, which give a more detailed display of the magnitude of changes in contrast to the measure of statistical significance (Fig. 2).

In ileum at 12 hpi, only three genes were significantly upregulated (FC > 1.5). *RNF213* is related to class I MHC-mediated antigen processing and presentation pathways and adaptive immune system pathways. *TRANK1* is a protein-coding gene, and its GO annotations for molecular functions include protein binding and ATP (Adenosine Triphosphate) binding. *RNF213* and *TRANK1* have orthologues in humans. *NCKAP5* is involved in the formation and modification of the microtubules.

At 24 hpi, the number of gene expression changes in ileum increased substantially (Fig. 1B). Ninety genes had significant fold-changes (76 genes upregulated, and 14 genes downregulated) (FDR < 5% and FC > 1.5; Table 1). The fold-change of gene expression between infected and non-infected groups ranged from 2.38 to − 1.41. The gene with the greatest significant upregulation was the early endosome antigen 1-like, a novel lncRNA. The significant upregulation of *RNF213* and *TRANK1* continued from 12- to 24-hpi time point. There was also augmented expression of antiviral factors, including innate immune responses, IFN-inducible genes and ISGs (*IFIT5, IFI6, IFIH1* (*MDA5*), *IFITM5*, *IFI27L2*, *STAT1, MX1, DHX58, DDX60,* and *OASL*). The *RSAD2* gene was also one of the top upregulated genes. GO terms for *RSAD2* were related to (1) defence responses to viruses, (2) CD4^+^ αβT cell activation and differentiation, (3) negative regulation of protein secretion and viral genome replication and (4) positive regulation of Toll-like receptor (TLR) 7/9 signaling pathways and T-helper 2 cell cytokine production. The *ZBTB32* gene was also among the significantly upregulated genes; its GO terms were related to negative regulation of transcription by RNA polymerase II. The *ZBTB32* orthologue in humans is *ZBTB16*, which is involved in the adaptive immune system and class I MHC-mediated antigen processing and presentation pathways. Heat-shock protein genes were also among the top results of upregulated genes, including *HSPH1, HSPA2, HSP90AA1*, *HSPB9*, *HSPA4L* and *HSPA8*. The *C1R* gene was also upregulated. It is involved in complement activation and the classical antibody-mediated complement activation pathway. *TLR3* was upregulated in this study. This gene encodes TLR 3, which recognizes double-stranded RNA and plays a vital role in pathogen recognition and activation of innate immunity.

Among the significantly downregulated genes in ileum at 24 hpi were *GNLY*, *CD3D*, *CST7*, *PTPN7*, *WAS* and *XCL1*. *GNLY*, *CD3D* and *CST7* are involved in the innate immune system. The *PTPN7* annotation was cytokine signaling in the immune system, and *WAS* is involved in the Fc-gamma receptor-dependent phagocytosis pathway. *XCL1* is involved in chemokine-mediated signaling pathways, G protein-coupled receptor signaling pathway, and cellular responses to IFN-gamma, IL-1 and tumour necrosis factor.

Of the 37 DE genes in ileum of infected chickens at 72 hpi, 25 genes had significant fold-changes (Table 1). *HSP*s were in the top results (*HSPH1* (HSP110), *HSP90AA1* (HSP90), *HSPA4L*, *HSPA5* and *HSPA8* (HSP70)). Additionally, *PDK4, CHAC1, DNAJA4* and *DNAJA1* were upregulated. In mice with severe influenza, inhibition of PDK4 using a therapeutic agent results in prevention of multiorgan failure^39^. *CHAC1* gene annotations included the intrinsic apoptotic signaling pathway in response to endoplasmic reticulum stress, which usually results from the accumulation of unfolded or misfolded proteins in the lumen of the endoplasmic reticulum lumen (GO:0,070,059), and negative regulation of the Notch signaling pathway (GO:0,045,746). Like cecal tonsils results, *DNAJA4* and its paralog *DNAJA1* gene were also upregulated in ileum at 72 hpi. The *DNAJA4* gene has related annotations, including unfolded protein binding and HSP binding (GO:0,051,082, GO:0,031,072). *TGIF1* gene was upregulated and involved in signaling by the transforming growth factor (TGF)-β receptor complex pathway.

The genes that were most downregulated in ileum at 72 hpi included *P2RY4*, which is involved in the G protein-coupled receptor signaling pathway. *ELF3* was also downregulated, and one of its gene ontogeny biological processes terms was inflammatory response to infection (GO:0,006,954). ELF1 is a protein encoded by the *ELF1* gene, which is a paralog of *ELF3* gene and regulates broad antiviral responses distinct from the type I IFN response^40^.

### Functional annotation analysis

#### Functional analysis of Reactome pathways of DE genes

Functional annotation analysis of Reactome pathways using the PANTHER overrepresentation test among significantly upregulated genes resulted in three statistically significant (FDR < 5%, Fisher’s exact test) Reactome pathways in cecal tonsils and ileum at 24 hpi. In both tissues, the highest enriched pathway was the ISG15 antiviral mechanism (Table 2).

**Table 2:** Functional annotation analysis of Reactome pathways using PANTHER.

Reactome pathways	Number of genes in reference list	Number of genes in target list	Expected	Over/under representation (+ /-)	Fold enrichment	Raw P-value	FDR
**Cecal tonsils at 24 h post-infection**							
ISG15 antiviral mechanism (R-GGA-1169408)	25	6	0.11	+	55.39	3.57E−09	5.58E−06
Antiviral mechanism by IFN-stimulated genes (R-GGA-1169410)	27	6	0.12	+	51.28	5.34E−09	4.17E−06
Interferon Signaling (R-GGA-913531)	49	6	0.21	+	28.26	1.30E−07	6.75E−05
**Ileum at 24 h post-infection**							
ISG15 antiviral mechanism (R-GGA-1169408)	25	5	0.1	+	51.51	9.88E−08	1.54E−04
Antiviral mechanism by IFN-stimulated genes (R-GGA-1169410)	27	5	0.1	+	47.69	1.39E−07	1.08E−04
Interferon Signaling (R-GGA-913531)	49	5	0.19	+	26.28	2.04E−06	1.06E−03

Overrepresentation analysis was performed on PANTHER. The analysis was performed on upregulated genes of significant gene changes in each tissue at each time point by filtering genes with a fold change of + /− 1.5 and significant *P*-value of False Discovery Rate (FDR) controlled at 5%. *IFN* interferon, *ISG* IFN-stimulated genes.

#### Functional analysis of gene ontology of DE genes

**Figure 3:**
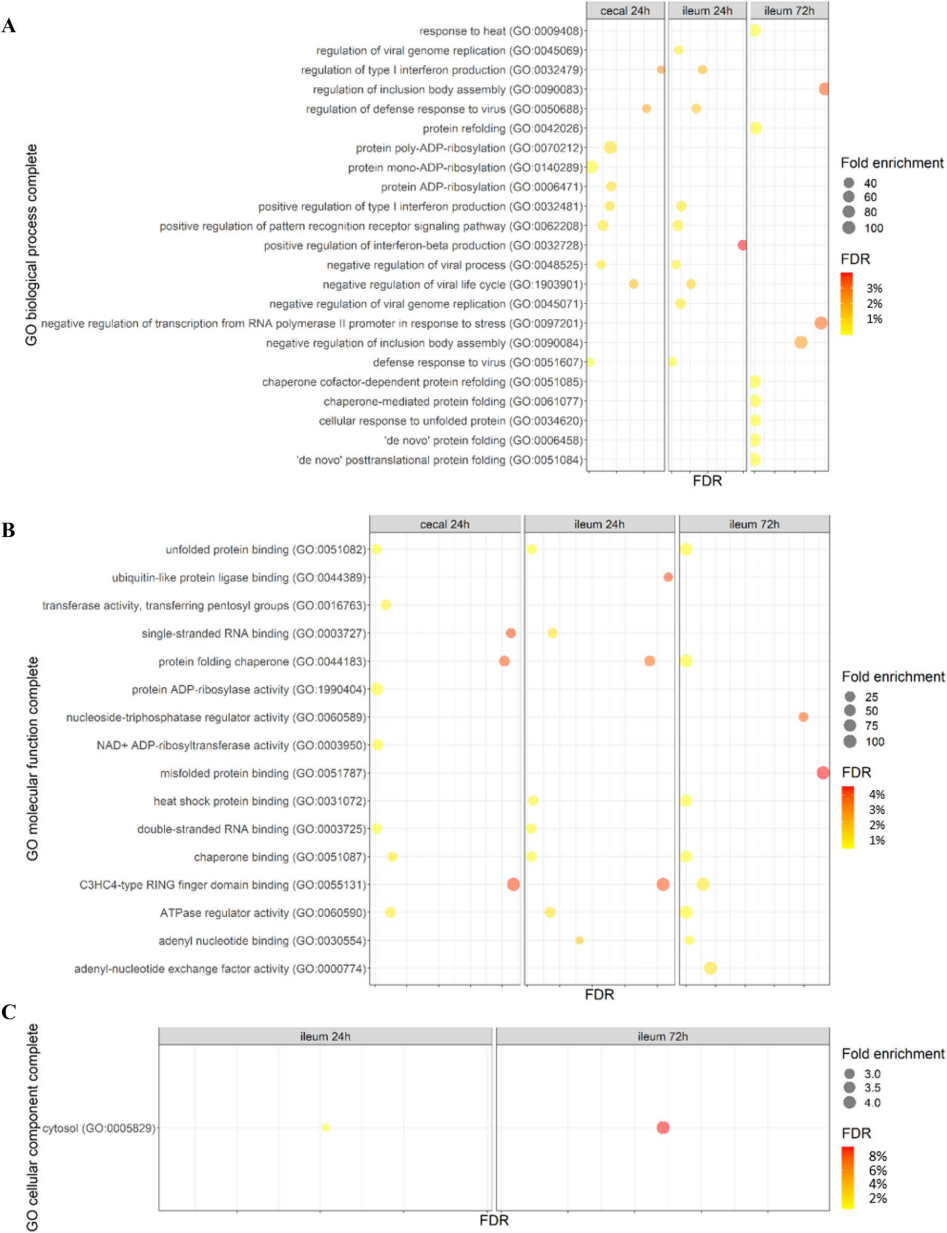
Dot plots of functional annotation analysis of gene ontology (GO) using PANTHER. Overrepresentation Test was performed on PANTHER among upregulated genes. Analysis was performed on gene list of each tissue at each time point by filtering for genes with a fold change of + /- 1.5 and False Discovery Rate (FDR) of < 5%. The top 10 GO terms of each tissue at each time point are plotted. The size of the dots represents the number of genes in the significant differentially expressed genes list associated with the GO term, and the colour of the dots represents the FDR values. (**A**) Biological Process. (**B**) Molecular function. (**C**) Cellular Components.

Functional annotation analysis of GO using the PANTHER overrepresentation test among upregulated genes gave results in cecal tonsils at 24 hpi and ileum at 24 and 72 hpi. In cecal tonsils at 24 hpi, the significantly upregulated genes were classified into 27 biological processes and 27 molecular functions with FDR of < 5% (Supplementary Tables 7 and 8) according to the GO project, respectively. The top 10 results of enriched biological processes are shown in Fig. 3A. The main GO categories of biological processes for the upregulated genes were protein ADP (Adenosine Diphosphate)-ribosylation, positive regulation of PRR signaling pathways, positive regulation of cytokine production (e.g. positive regulation of type I IFN production) and regulation of viral life cycle (e.g. negative regulation). The top 10 results of enriched molecular functions are shown in Fig. 3B. The GO terms of the molecular functions for the upregulated genes included protein folding chaperone, single-stranded RNA binding and HSP binding.

In ileum at 24 hpi, the upregulated genes were classified into 30, 21 and one functional groups (FDR < 5%) of biological processes, molecular functions and cellular components, respectively (Supplementary Tables 9, 10 and 11). The top 10 results of enriched biological processes are shown in Fig. 3A. The main GO categories of the biological processes were regulation of PRR signaling pathways, positive regulation of cytokine production (e.g. IFN-β), negative regulation of viral process (e.g. negative regulation of viral genome replication) and response to stress (e.g. defense response to virus). The top 10 results of enriched molecular functions are shown in Fig. 3B. The GO terms of the molecular functions included protein folding chaperone, double- and single-stranded RNA binding, chaperone binding and unfolded protein binding. Figure 3C shows the cellular component (cytosol) as the only enriched GO term relevant to ileum at 24 hpi.

In ileum at 72 hpi, the DE upregulated genes were classified into 19 biological processes, 19 molecular functions and one cellular component at FDR < 5% (Supplementary Tables 12, 13 and 14) according to the GO project, respectively. The top 10 results of enriched biological processes are shown in Fig. 3A. The main biological processes were negative regulation of inclusion body assembly, negative regulation of transcription from RNA polymerase II promoter in response to stress and protein refolding. Figure 3B presents the top 10 enriched molecular functions, including C3HC4-type RING (Really Interesting New Gene) finger domain binding, protein folding chaperone, misfolded protein binding, chaperone binding and HSP binding. The cellular component (cytosol) was the only enriched GO term in ileum at 72 hpi (Fig. 3C).

## Discussion

Immunity against AIV is the result of an orchestrated effort between innate and adaptive immune responses to this virus. Transcriptomic studies have been carried out in the GIT of chickens during infection with various subtypes of influenza A viruses. Infection with H5N1 or H5N2 in chickens results in differential expression of genes associated with innate immune responses in ileum^8^. Also, transcriptome analysis of ileum from birds infected with high (H5N1) and low (H5N2) pathogenic influenza viruses has revealed very different *IFITM* responses in ducks and chickens^31^. Yet, there is no comprehensive understanding of the mechanisms of immune response to LPAIV, such as H9N2, in the GIT. The present study was designed to evaluate the global gene expression changes in cecal tonsils and ileum of chickens after infection with H9N2 AIV. The induced expression patterns of different genes in ileum and cecal tonsils of AIV-infected chickens were revealed with some important observations.

Comparing the gene expression profiles of cecal tonsils and ileum, we observed some shared and unique responses to AIV infection. At 24 and 72 hpi, the comparison of differentially expressed genes showed that there were no genes displaying an opposite expression pattern between the two tissues and between the time points post-infection (Supplementary Fig. 2A-D). None of the sets resulted in any pathway with FDR <  = 5%, except the upregulated genes in ileum at 24 hpi. The most enriched pathways were the regulation of gene expression by hypoxia-inducible factor (HIF) pathway (R-GGA-1234158) and the TRAF6-mediated interferon regulatory factor (IRF7) activation pathway (R-GGA-933541). The mechanism of viral activation of the HIF-1 pathway is that influenza A virus infection causes impaired proteasome function, resulting in HIF-1α accumulation, and decreases expression of factor inhibiting HIF-1, further boosting HIF-1 activity^41^. Nuclear translocation of HIF-1α induced by influenza A infection is crucial to pro-inflammatory cytokines production, and deficiency of HIF-1α in lung epithelial cells enhances autophagy, facilitating influenza A virus replication^42,43^. Activation of IRF7 is triggered by TLR7 recognition of RNA released from the influenza virus, which is followed by the production of type I IFNs. Moreover, TRAF6-mediated IRF7 activation establishes innate immune responses and the absence of TRAF6 ensues enhanced viral replication and a significant reduction in IL-6 and type I IFNs production after infection with RNA virus^44^.

In the present study, *RNF213* was upregulated gene in ileum at 12 and 24 hpi and cecal tonsils at 24 hpi. The *Rnf213* gene was identified as highly expressed among a cluster of ISGs with known antiviral activity in a mouse model^45^. The model revealed that the heterogeneity of influenza replication from cell to cell is not derived by cell type. Also, the cells with no or low viral replication have high induction of the *Rnf213* gene^45^. Additionally, a low abundance of virus transcripts is associated with the host innate response (response to type I IFN) gene cluster, including the *RNF213* gene, which is significantly highly expressed in response to influenza infection^46^. Further, a transcriptomic analysis has revealed new genes and networks in response to H5N1 infection in ducks; among them, the *RNF213* gene is expressed differentially in the brain post-infection^47^.

Here, we report that a novel gene called early endosome antigen 1-like and identified as a lncRNA had significant upregulation in cecal tonsils and ileum at 24 hpi. Although little is known about lncRNAs, a lncRNA is upregulated due to viral delivery and is required to induce antiviral effects by promoting an increase in IFN production^48^. Furthermore, an evolutionarily conserved role for lncRNAs has been suggested, as conserved sequences are found to define virus-specific effector and memory CD8^+^ T cells^49^. Early endosome antigen 1 (*EEA1*), a protein-coding gene, is involved in vesicular trafficking and is affected by bacterial infections^50,51^. Intracellular pathogens target molecular factors, including *EEA1*, and inhibit its recruitment, triggering phagosomal maturation arrest^50,52^. Influenza A viruses benefit from *EEA1* to infect human cells; following internalization of virus particles in dendritic cells, the influenza virus traffic through EEA1^+^ early endosomes to gain access to the cytoplasm^53^. Chicken *EEA1* is a coding orthologue of human *EEA1.* It is hypothesized that lncRNA is involved in competing for endogenous RNA (ceRNA), which could regulate a protein-coding gene by sponging up microRNA^54^. The infected cells may increase early endosome antigen 1-like expression to limit viral entry to cells in the current study.

DExD/H-box helicases, such as *DDX3*, have been involved as sensors in innate antiviral immunity^55–58^. *DDX60* is involved in RIG-I-dependent and independent antiviral responses^59^. Several DEAD/DEAH-box RNA helicases are hijacked by RNA viruses in infected cells to facilitate their replication^60–64^. Chicken *DDX60* and *DHX58* have orthologues in humans. In the present study, two IFN-inducible cytoplasmic helicases were upregulated in chicken GIT post-infection: *DDX60* (DExD/H-box helicase 60) and *DHX58* (DExH-box helicase 58, also known as LGP2 (laboratory of genetics and physiology 2)). Our observations supported the role of DExD/H-box helicases in innate antiviral immunity and their manipulation by viruses. *DDX60* upregulation started at 12 hpi in ileum but not in cecal tonsils, which then reached significant upregulation at 24 hpi in both tissues, and then subsided at 72 hpi. This gene expression may imply a broader function of chicken DExD/H-box helicases and provide a deeper understanding of immune mechanisms and pathogenesis of AIV in chicken GIT.

Heat shock proteins shared across Archea, bacteria and eukaryotes, are associated with cellular injury. Expression of HSPs is elevated to protect cells under stress and is involved in protein folding, the immune system and antiviral response. However, viruses lack HSPs and depend on host HSPs for their protein folding^65^. Therefore, viruses can hijack HSPs, including hsp40, hsp70 and hsp90, to increase viral replication^66^. Exogenous administration of HSPs can enhance immune responses in cancer and infectious diseases by activation of protective T cell responses^67,68^. HSPs can also interact with various viral proteins; for example, HSPA8 can interact with influenza virus protein M1. This interaction inhibits the nuclear export of matrix protein 1 and nucleoprotein and subsequently inhibits viral assembly^69^. Upregulation of HSPs has been reported in the chicken respiratory tract^27^. In the present study, we found that HSPs were upregulated significantly at 24 and 72 hpi in ileum and cecal tonsils of AIV-infected chickens. Targeting HSPs could provide a platform for prophylactic or therapeutic strategies to manipulate immune responses against AIV in poultry.

Viruses also exploit cytoskeleton proteins during early viral replication^70,71^. TCP1 is a chaperonin and a cytosolic HSP60 family protein^72^. The gamma subunit of TCP1 plays a vital role in the folding and assembling of cytoskeleton proteins^73,74^. Chicken *TCP1* has a human orthologue—section of the gene that codes for the gamma-like subunit. T-complex protein 1 subunit gamma-like, a novel gene in the chicken genome, is a paralogue of chicken *TCP1*. In the present study, we found the most upregulation of the T-complex protein 1 subunit gamma-like gene in cecal tonsils at 24 hpi. This unique gene upregulation in cecal tonsils may signal this gene's employment by H9N2 AIV in the current study for its purposes in protein folding and assembly.

We found that *MX1* gene was upregulated in ileum and cecal tonsils. This may draw a link to an anti-viral response in chicken cecal tonsils to AIV infection. MX1 protein inhibits the import of nuclear material into viral nucleocapsids, which is known to have a disruptive effect on influenza A viruses by disrupting lipid rafts of the virus. Other transcriptomic studies in poultry have also shown upregulation of this gene in repertory tract and ileum^8,24,27^.

IFITM is a family of genes known to have a role in limiting influenza infection^75^. Transcriptomic analysis shows that chickens mount a weak *IFITM* response in ileum post-infection with LPAIV H5N2^31^. However, the findings of our study showed upregulation of *IFITM5* in cecal tonsils and ileum at 24 hpi. This discrepancy of *IFITM* response may be because of the different subtypes used.

The results of the functional analyses of the DE genes were related to the immune responses to influenza infection. GO terms and Reactome pathways results were almost exclusively associated with response to viral infection. In cecal tonsils and ileum at 24 hpi, one of the highest enriched pathways was the ISG15 antiviral mechanism (Supplementary Fig. 1). IFN-induced ISG15 targets the non-structural 1 (NS1A) protein of the influenza A virus, causing a loss of function of the NS1A protein and the inhibition of influenza A virus replication in virus-infected cells^76^. Additionally, ISG15 plays various roles in the defence against viral infections and the activation of immune system cells such as lymphocytes, monocytes, and natural killer cells^77^. Interestingly, the NS1A protein of influenza A viruses is responsible for inhibiting host immune responses by limiting production of IFNs and, therefore, antiviral effects of IFN-induced proteins^78^.

In conclusion, our study illustrated the differential expression of several innate immune system genes following AIV infection in ileum and cecal tonsils. The results of the present study revealed that there is some degree of similarity between immune responses in GIT and those induced in the respiratory tract, although further research is required to determine whether there are spatial and temporal differences in gene expression between the gastrointestinal and respiratory tracts. Our study highlighted some of the DE genes that are known to be exploited by the influenza virus to support viral replication. Some of the DE genes have been consistently identified in chicken LPAIV studies, hinting that they may be the best candidates for future research. One important finding was the enriched antiviral pathway of ISG15. ISG15 plays a critical role in innate responses, and future research studying this mechanism might provide a platform for strategies against AIV in poultry.

## Materials and methods

### Experimental design

This study included two main factors: treatment (challenged versus nonchallenged) and time (three time points post-infection), resulting in six groups. Each group contained six biological replicates. A total of 36 one-day-old specific pathogen-free White Leghorn layers were purchased from the Canadian Food Inspection Agency (Ottawa, Canada) and housed in the isolation facility at the Ontario Veterinary College at the University of Guelph. All experimental procedures were approved by the University of Guelph Animal Care Committee and conducted according to specifications of the Canadian Council on Animal Care. Experiments in the manuscript followed the recommendations in the ARRIVE guidelines.

Twenty-one days after hatching, 18 chickens in the challenged group were inoculated via oculo-nasal and oral routes with 250 μL of 3.1 × 10^7^ TCID_50_/mL of LPAIV H9N2. Eighteen chickens in the nonchallenged group were inoculated via the same route with 200 μL of phosphate-buffered saline. The ileal samples at proximal ileocecal transition and cecal tonsils were collected from six chickens per time point at 12, 24, and 72 h post-infection (hpi) per treatment. The collected samples were transferred into tubes containing 1 mL of RNAlater solution (Invitrogen, Burlington, ON, CAN) for RNA stabilization and storage for later processing. The above time points were chosen that coincides with induction of innate responses to the virus.

### Virus strain and virus propagation

A/TK/IT/13VIR1864-45/2013 (H9N2) from the Istituto Zooprofilattico Sperimentale delle Venezie (IZSVe), Italy, was used in this study. The virus was propagated in 10-day-old embryonated specific pathogen-free chicken eggs. After 72 h of incubation at 37 °C, eggs were refrigerated at 4 °C overnight. Allantoic fluid was collected and purified by centrifuging at 400 × g for 5 min at 4 °C and then stored at -80 °C until further use. The propagated virus titer was determined with 50% tissue culture infectious dose (TCID_50_) on Madin-Darby canine kidney (MDCK) cells.

### Extraction, library preparation and RNA sequencing

Total RNA was extracted using the RNeasy Mini kit (Qiagen, Toronto, ON, CAN) according to the manufacturer’s instructions. Library construction, quality control testing and sequencing were performed by Novogene (Sacramento, USA). Briefly, quantification and quality control testing were performed on 1% agarose gels, the NanoPhotometer spectrophotometer (IMPLEN, CA, USA) and the RNA Nano 6000 Assay Kit in a Bioanalyzer 2100 system (Agilent Technologies, CA, USA). A total amount of 1 μg of RNA per sample was used for library preparation using a NEBNext Ultra RNA Library Prep Kit for Illumina (New England Biolabs, USA). Library quality was assessed on the Agilent Bioanalyzer 2100 system. The clustering of the index-coded samples was performed on a cBot Cluster Generation System using a PE Cluster Kit cBot-HS (Illumina). cDNA libraries were sequenced on an Illumina HiSeq-4000 platform, and 150 base pair paired-end reads were generated.

### RNAseq data analysis interpretation

RNA-seq analysis was performed on the Trimmomatic-STAR-RSEM-DESeq2 pipeline previously established^79^. Raw reads were processed for quality control. Clean data were obtained based on Phred score (a quality measure to assess the accuracy of a sequencing) by trimming adapters and nucleotides with a Phred score of less than 30 at the beginning and end of the reads and discarding nucleotides with a Phred score of less than 20 in the window of five nucleotides using Trimmomatic^80^. The minimum length of surviving reads was set at 36 nucleotides. All downstream analyses were based on clean data with high quality.

Reads were mapped to a Gallus gallus reference genome (GRCg6a, release 98) using Spliced Transcripts Alignment to a Reference (STAR) (version 2.7.0a)^81^. The number of reads per gene was quantified by RNA-Seq by Expectation–Maximization (RSEM)^82^. RSEM output files were used in DESeq2 (version 1.24.0) to calculate differentially expressed (DE) genes by comparing challenge group versus control group for each tissue per time point^83^. Genes with an absolute fold-change (FC) of more than a 1.5 (|FC|> 1.5; |log_2_FC|> 0.584) and with a significant p-value when False Discovery Rate (FDR) was controlled at 5% were considered DE. VennDiagram package in R was used to compare DE genes between cecal tonsils and ileum at 24 and 72 h time points^84^.

### Functional annotation analysis of DE genes

Overrepresentation analysis was performed on PANTHER (version 15) (http://www.pantherdb.org/) using the PANTHER Overrepresentation Test (Released 20,200,728) on DE genes of each tissue at each time point. Gene ontology (GO) database https://doi.org/10.5281/zenodo.3954044 Released 2020–07-16 (http://www.geneontology.org) was used to analyze the overrepresentation of GO terms in all gene lists. Reactome pathways (from Reactome database version 65, Released 2019–12-22) was used to analyze the overrepresentation of pathways among genes in all gene lists (https://reactome.org/). Both tests were performed using Fisher's exact test with FDR correction.

## Supplementary Information

Supplementary Information 1.

Supplementary Information 2.

Supplementary Information 3.

Supplementary Information 4.

## Data Availability

The datasets generated and analyzed during the current study are available in SRA database, BioProject ID PRJNA735215.
