# Supplementary material for: Transcriptomics of chicken cecal tonsils and intestine after infection with low pathogenic avian influenza virus H9N2
Source: Sci Rep. 2021 Oct 14;11:20462. doi: 10.1038/s41598-021-99182-3 (PMC8517014; doi:10.1038/s41598-021-99182-3)
Supplement: Supplementary file 3 — Supplementary Information 3. [file 41598_2021_99182_MOESM3_ESM.pdf]

Transcriptomics of chicken cecal tonsils and intestine after infection with low pathogenic avian influenza virus H9N2

Nadiyah Alqazlan[1], Mehdi Emam[2], Éva Nagy[1], Byram Bridle[1], Mehdi Sargolzaei[1,3], and Shayan Sharif[1,*]

[1]Department of Pathobiology, Ontario Veterinary College, University of Guelph, Guelph, Ontario N1G 2W1, Canada
[2]Department of Human Genetics, McGill University, Montreal, Quebec H3A 0E7, Canada´
[3]Select Sires, Inc. Plain City, OH 43064, United States [*]corresponding.shayan@uoguelph.ca

Supplementary figure 1. The ISG15 pathway. There are six upregulated genes (listed in the table) with a role in the ISG15 pathway. A red dotted line indicates them in the graph.

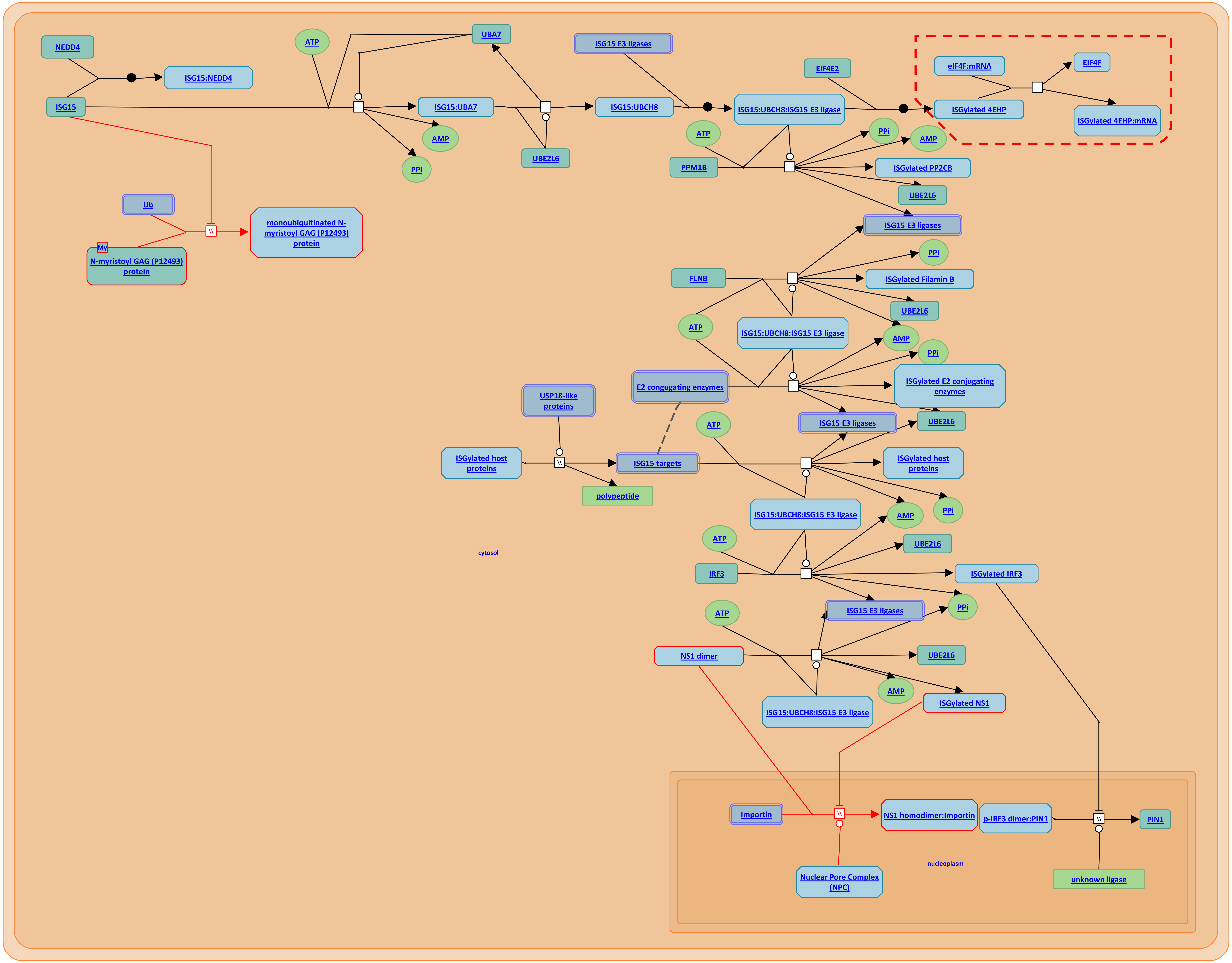

NEDD4
ATP
UBA7
ISG15 E3 ligases
EIF4E2
eIF4F:mRNA
EIF4F
ISG15:NEDD4
ISG15
ISG15:UBA7
ISG15:UBCH8
ISG15:UBCH8:ISG15 E3 ligase
ISGylated 4EHP
ISGylated 4EHP:mRNA
AMP
PPi
UBE2L6
ATP
PPi
AMP
PPM1B
ISGylated PP2CB
UBE2L6
ISG15 E3 ligases
Ub
My
monoubiquitinated N-myristoyl GAG (P12493) protein
N-myristoyl GAG (P12493) protein
PPi
FLNB
ISGylated Filamin B
UBE2L6
ATP
ISG15:UBCH8:ISG15 E3 ligase
AMP
PPi
E2 congugating enzymes
ISGylated E2 conjugating enzymes
USP18-like proteins
ISG15 E3 ligases
UBE2L6
ATP
ISGylated host proteins
ISG15 targets
ISGylated host proteins
polypeptide
ISG15:UBCH8:ISG15 E3 ligase
AMP
PPi
cytosol
ATP
UBE2L6
IRF3
ISGylated IRF3
ISG15 E3 ligases
PPi
ATP
NS1 dimer
UBE2L6
AMP
ISGylated NS1
ISG15:UBCH8:ISG15 E3 ligase
Importin
NS1 homodimer:Importin
p-IRF3 dimer:PIN1
PIN1
nucleoplasm
Nuclear Pore Complex (NPC)
unknown ligase

Upregulated genes in cecal tonsils at 24 hours post-infection with a role in ISG15 pathway

	Gene ID	Mapped IDs	Gene Name	PANTHER Family/Subfamily
			**Gene Symbol**	
**1**	CHICK|Ensembl=ENSGALG00000016142|UniProtKB=B2X021	ENSGALG00000016142	Mx protein	INTERFERON-INDUCED GTP-BINDING PROTEIN MX1 (PTHR11566:SF217)
			Mx	
**2**	CHICK|Ensembl=ENSGALG00000007651|UniProtKB=Q5ZJK3	ENSGALG00000007651	Signal transducer and activator of transcription	SIGNAL TRANSDUCER AND ACTIVATOR OF TRANSCRIPTION 1-ALPHA/BETA (PTHR11801:SF18)
			STAT1	
**3**	CHICK|Ensembl=ENSGALG00000003144|UniProtKB=A0A0B5H7I8	ENSGALG00000003144	Tripartite motif containing 25	E3 UBIQUITIN/ISG15 LIGASE TRIM25 (PTHR25465:SF17)
			TRIM25	
**4**	CHICK|Ensembl=ENSGALG00000045085|UniProtKB=A0A1L1RTT2	ENSGALG00000045085	Uncharacterized protein	TRANSCRIPTION ACTIVATOR GUTR (PTHR10271:SF0)
			IFIT5	
**5**	CHICK|Ensembl=ENSGALG00000010560|UniProtKB=F1NLD7	ENSGALG00000010560	Uncharacterized protein	INTERFERON-INDUCED, DOUBLE-STRANDED RNA-ACTIVATED PROTEIN KINASE (PTHR11042:SF163)
			EIF2AK2	
**6**	CHICK|Ensembl=ENSGALG00000013057|UniProtKB=E1BXQ0	ENSGALG00000013057	Ubiquitin carboxyl-terminal hydrolase	UBL CARBOXYL-TERMINAL HYDROLASE 18-RELATED (PTHR24006:SF796)
			USP41	
