# Supplementary material for: Transcriptomics of chicken cecal tonsils and intestine after infection with low pathogenic avian influenza virus H9N2
Source: Sci Rep. 2021 Oct 14;11:20462. doi: 10.1038/s41598-021-99182-3 (PMC8517014; doi:10.1038/s41598-021-99182-3)
Supplement: Supplementary file 4 — Supplementary Information 4. [file 41598_2021_99182_MOESM4_ESM.pdf]

Transcriptomics of chicken cecal tonsils and intestine after infection with low pathogenic avian influenza virus H9N2

Nadiyah Alqazlan[1], Mehdi Emam[2], Éva Nagy[1], Byram Bridle[1], Mehdi Sargolzaei[1,3], and Shayan Sharif[1,*]

[1]Department of Pathobiology, Ontario Veterinary College, University of Guelph, Guelph, Ontario N1G 2W1, Canada
[2]Department of Human Genetics, McGill University, Montreal, Quebec H3A 0E7, Canada´
[3]Select Sires, Inc. Plain City, OH 43064, United States [*]corresponding.shayan@uoguelph.ca

Supplementary figure 2. Venn diagrams showing shared and unique responses to AIV infection in cecal tonsils and ileum. Responses to H9N2 AIV infection in ileum is compared to that in cecal tonsils for upregulated genes at 24 hpi (A) and at 72 hpi (B) and for downregulated genes at 24 hpi (C) and at 72 hpi (D).

A.

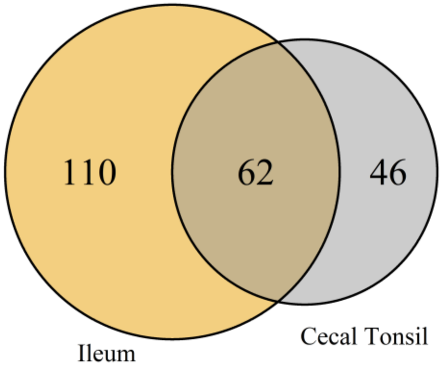

Upregulated genes at 24hpi

B.

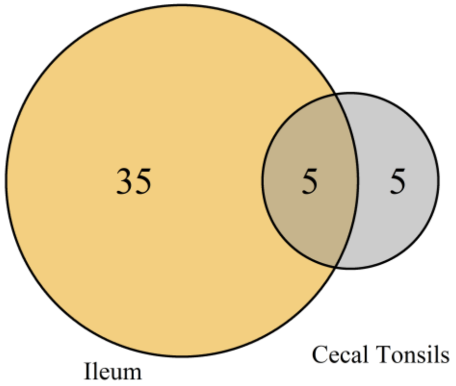

Upregulated genes at 72hpi

C.

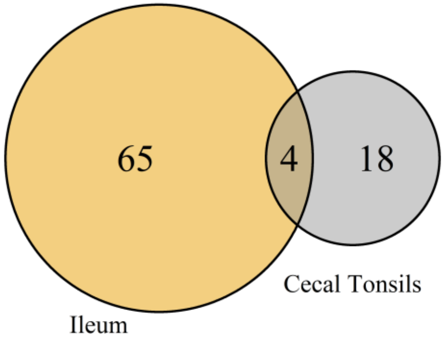

Downregulated genes at 24hpi

D.

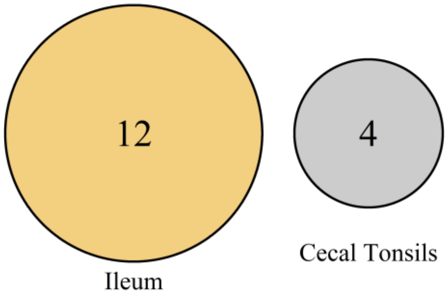

Downregulated genes at 72hpi

Functional annotation analysis of Reactome pathways using PANTHER

Overrepresentation analysis was performed on PANTHER. The analysis was performed on 110 genes exclusively upregulated in ileum at 24 hpi by filtering genes with a significant p-value of False Discovery Rate (FDR) controlled at 10%.

**Reactome pathways**	**Number of genes in reference list**	**Number of genes in target list**	**Expected**	**Over/under representation (+/-)**	**Fold enrichment**	**Raw P-value**	**FDR**
Regulation of gene expression by Hypoxia-inducible Factor (R-GGA-1234158)	3	2	0.02	+	> 100	1.32E-04	6.73E-02
TRAF6 mediated IRF7 activation (R-GGA-933541)	3	2	0.02	+	> 100	1.32E-04	5.05E-02
Formation of the beta-catenin:TCF transactivating complex (R-GGA-201722)	12	3	0.07	+	45.63	4.38E-05	6.68E-02
Transcriptional regulation by the AP-2 (TFAP2) family of transcription factors (R-GGA-8864260)	13	3	0.07	+	42.12	5.54E-05	4.23E-02
SUMO E3 ligases SUMOylate target proteins (R-GGA-3108232)	88	5	0.48	+	10.37	1.34E-04	4.09E-02
SUMOylation (R-GGA-2990846)	90	5	0.49	+	10.14	1.48E-04	3.78E-02
